# Unveiling Tumor Microenvironment Interactions Using Zebrafish Models

**DOI:** 10.3389/fmolb.2020.611847

**Published:** 2021-01-14

**Authors:** Reid Loveless, Chloe Shay, Yong Teng

**Affiliations:** ^1^Department of Oral Biology and Diagnostic Sciences, Dental College of Georgia, Augusta University, Augusta, GA, United States; ^2^Department of Pediatrics, Emory Children's Center, Emory University, Atlanta, GA, United States; ^3^Georgia Cancer Center, Department of Biochemistry and Molecular Biology, Medical College of Georgia, Augusta University, Augusta, GA, United States; ^4^Department of Medical Laboratory, Imaging and Radiologic Sciences, College of Allied Health, Augusta University, Augusta, GA, United States

**Keywords:** zebrafish, tumor escape, preclinical models, tumor microenvironment, tumor support

## Abstract

The tumor microenvironment (TME) is a rich and active arena that is strategically evolved overtime by tumors to promote their survival and dissemination. Over the years, attention has been focused to characterize and identify the tumor-supporting roles and subsequent targeting potentials of TME components. Nevertheless, recapitulating the human TME has proved inherently challenging, leaving much to be explored. In this regard, *in vivo* model systems like zebrafish, with its optical clarity, ease of genetic manipulation, and high engraftment, have proven to be indispensable for TME modeling and investigation. In this review, we discuss the recent ways by which zebrafish models have lent their utility to provide new insights into the various cellular and molecular mechanisms driving TME dynamics and tumor support. Specifically, we report on innate immune cell interactions, cytokine signaling, metastatic plasticity, and other processes within the metastatic cascade. In addition, we reflect on the arrival of adult zebrafish models and the potential of patient-derived xenografts.

## Background

The tumor microenvironment (TME) has been appreciated for its principal role in tumor development and progression as well as therapeutic resistance for quite some time. Composed of proliferating tumor cells, stromal cells, like endothelial cells, fibroblasts, and immune cells, vasculature, and the extracellular matrix (ECM), the TME maintains remarkable molecular and cellular heterogeneity and can be further characterized by hypoxia, acidosis, and ECM stiffness. Although communication within the TME occurs bilaterally, tumors functionally modulate, and exploit their microenvironment through various molecular mechanisms and opportune relations to confer their survival and dissemination (Miranda-Galvis and Teng, 2020). Here, processes like ECM remodeling, neo-angiogenesis, and the corruption of resident and infiltrating cells occur regularly in order to develop fertile “soils.”

**Figure 1:**
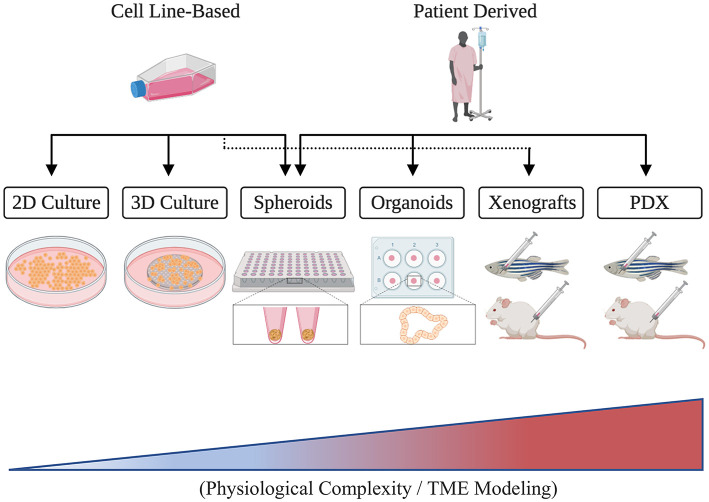
A schematic illustrating different cancer modeling methods in the order of increasing tumor microenvironment (TME) representation.

The TME's fundamental contributions to the six cancer hallmark capabilities established by Hanahan and Weinberg (2000, 2011) underscore its grave implications and translational significance. Unveiling the genetic and molecular components governing TME interactions, therefore, has become the cardinal focus for many cancer researchers who aim to identify novel therapeutic targets and develop effective patient therapies (Letrado et al., 2018; Roma-Rodrigues et al., 2019). Nonetheless, much remains to be understood regarding the individual and combined functions of TME constituents and the ways by which they communicate. While 2D and 3D *in vitro* assays have proven indispensable for the controlled investigation of particular aspects of the TME, they, unfortunately, remain limited in their capacity to capture the combined pathophysiological and phenotypical complexity that is present clinically (Figure 1) (Hoarau-Véchot et al., 2018). In consequence, *in vivo* model organisms like mice and zebrafish have been used in order to achieve more faithful recapitulation and generalizability.

In this review, we attempt to highlight the recent ways zebrafish models have contributed to our understanding of the TME interactions promoting tumor progression. First, we highlight zebrafish's intrinsic investigative capacity then transition our focus onto the TME within the context of the immune system and later within the metastatic cascade. Lastly, we discuss the employment of adult zebrafish models and the potential utility of zebrafish patient-derived xenografts (zPDX).

## Zebrafish Possess Attractive Advantages to Probe Tumor Microenvironment Interactions

Given their anatomical and genetic similarities to humans, murine models have been used to study cancer for over a century. Like all model systems, however, their intrinsic features restrict their investigative utility. For example, because of their fur, they are largely incompatible with intravital imaging (with the exception of implanted imaging windows; Day et al., 2015) and, thus, do not permit active and reliable monitoring of TME interactions throughout the metastatic cascade. Moreover, in the context of husbandry, mice are relatively expensive, require moderate time and space to develop, and do not possess high-throughput capabilities. Zebrafish, on the other hand, with its optical clarity, high fecundity, and ease of genetic manipulation, has emerged as a potent model system to visualize cancer biology at single-cell-level resolution (Table 1). Although its genetic similarity to humans (70%) is less than that of mice to humans (80%), it nonetheless maintains high conservation of genes and signaling pathways (Teng et al., 2010, 2011), especially those involved in vertebrate cancer. Larval or immunocompromised adult zebrafish also readily engraft human cancer cells, which have been seen to have similar growth kinetics and histology when compared with those grown in mice (Yan et al., 2019). Moreover, we have shown that zebrafish tumor xenografts can be used to faithfully evaluate the metastatic potential of human cancer cell lines and primary tumors, permitting robust metastatic evaluation of single-gene mutations (Teng et al., 2013; Xie et al., 2015). Taken alongside its low cost, high speed, and high throughput capacity, zebrafish has secured its reputation as a well-poised model for basic research and can be seen preparing for its entrance into the arena of personalized medicine.

**Table 1:** Employable imaging techniques for the investigation of TME interactions in zebrafish.

**Technique**	**Applications**	**Penetration**	**Disadvantages**	**References**	**Example**
Stereomicroscopy	Possesses potential for fluorescent and time-lapse imaging (live and fixed)	Not limited	Requires transparent fish	Paatero et al., 2018	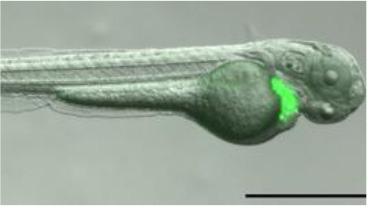
Conventional confocal microscopy	3D imaging and time lapses (live and fixed)	Up to 200 μm	Can be time-consuming	van den Berg et al., 2019	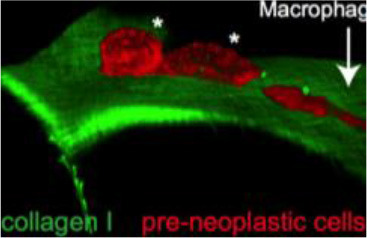
Correlative light and electron microscopy	Multimodal: 3D imaging with definition of ultrastructure (live and fixed)	Up to 200 μm	Time-consuming	van den Berg et al., 2019	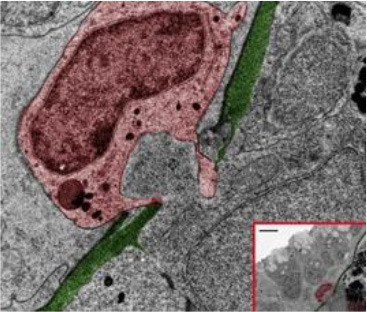
Two photon (multiphoton)	Cellular behavior and membrane order; commonly use fluorescent dyes or endogenous markers (live and fixed)	Up to 500 μm	Potential for thermal damage; decreased molecular brightness	Perrin et al., 2020	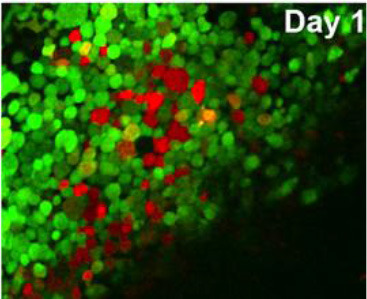
Second harmonic generation (multiphoton)	Non-centrosymmetric structures like collagen fibers (live and fixed)	Up to 300 μm	Limited applicability to structural proteins	LeBert et al., 2016	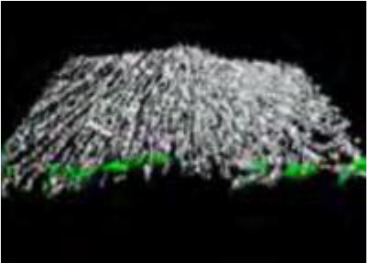
Selective plane illumination microscopy (light sheet)	3D imaging, deep optical sectioning (live and fixed)	Up to 3 mm	Extra optics required	Gualda et al., 2015	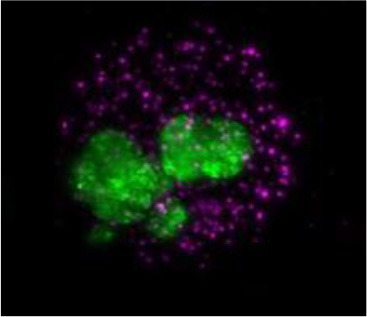
Micro-CT	3D whole-organism imaging; phenotypic and architectural (live and fixed)	Not limited	Time-consuming	Ding et al., 2019	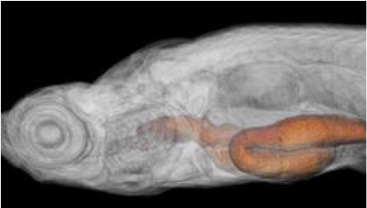

*TME, tumor microenvironment*.

## Zebrafish Facilitate Exploration Into Tumor-Immune Crosstalk

Under homeostatic conditions, innate (e.g., macrophages and neutrophils) and adaptive immunity (e.g., T cells and B cells) coordinately suppress tumor progression and prevent systemic perturbations. As tumor cells proliferate, however, they functionally distort the TME and disrupt tissue integrity, provoking an immune response that leads to the infiltration of immune cells and chronic inflammation (Comen et al., 2018). Although immunosurveillance can be used to identify and execute immunogenic cancer cells, mechanisms to elude detection and hijack immune defenses are quickly selected for and allow tumors to proliferate and disseminate alongside tumor-associated immune cells, like tumor-associated macrophages (TAMs) and tumor-associated neutrophils (TANs) (Ribas, 2015; Gonzalez et al., 2018). In addition to enticing immune dysregulation, tumor-immune interactions function to stimulate angiogenesis, enhance proliferation, and promote invasion, among other processes that fuel tumor progression (Blomberg et al., 2018).

Although the diverse and dynamic roles of immune cells throughout tumor progression remain to be fully elucidated, zebrafish have granted us a remarkable opportunity to investigate. In particular, because larval zebrafish lack functional adaptive immunity, they can be engrafted with human cancer and immune cells without fear of rejection. Importantly, they are also transparent at this stage, allowing engrafted cells to be closely and reliably monitored. Although larval zebrafish models' small size and development of functional adaptive immunity by 21 days post-fertilization do, respectively restrict tumor xenografts to 25–100 cells and prevent long-term engraftment studies, their ability to probe specific innate immune functions is underscored by their receptiveness to targeted gene mutation and transgene insertion (Moore and Langenau, 2016). In this regard, genome editing tools like CRISPR-Cas9 and Tol 2 system have not only allowed for robust reverse genetic screens and instrumental fluorescent reporter lines but give rise to a collection of genetic zebrafish cancer models though mutation of zebrafish orthologs and introduction of mutated human genes (Hason and Bartuněk, 2019). In consequence, the number of cancer studies employing zebrafish continues to rise alongside our understanding of the various ways by which immune components, directly, and indirectly, aid cancer cells throughout their progression.

### Interrogating the Pro-tumor Interactions of Innate Immune Cells

Elucidating the tumor-supporting roles of innate immune cells like macrophages and neutrophils has remained inherently challenging due to their dynamic and tissue-specific functions. Zebrafish provide a unique model system to monitor these cells' interactions while assessing their broader tumor-associated implications, however.

Particularly, it has been demonstrated that human macrophages and monocytes can survive unmolested for up to 2 weeks in zebrafish larvae at both zebrafish (28.5°C) and human (37°C) physiological temperatures (Paul et al., 2019b). Here, they have been seen to not only adopt active phenotypes but also undergo transformation by host cells like astrocytes (Paul et al., 2019b). Notably, the effects of intercellular communication between macrophages and tumor cells, specifically through long membranous tunneling nanotubes (TNTs), have recently been explored using zebrafish. Here, heterotypic TNTs were seen to enhance the invasive phenotype of tumor cells in an EGF-EGFR-dependent manner and to be critical to the directional streaming of tumor cells toward the endothelium (Hanna et al., 2019). Accordingly, disruption of macrophage TNT formation *in vivo* significantly reduces tumor invasion (Hanna et al., 2019). Communication between tumor cells and macrophages can also occur over greater distances via tumor-secreted extracellular vesicles (EVs) carrying pro-tumoral and pro-metastatic factors. Through chemical and transgenic probes, Hyenne et al. (2019) have developed a strategic approach to track the hemodynamic behavior and fate of circulating EVs in zebrafish. After circulation arrest, it was seen that EVs derived from a melanoma line (Zmel1) are largely taken up by endothelial cells and patrolling macrophages (Verweij et al., 2019), reducing macrophage motility and promoting the expression of TNF-α. Furthermore, these exogenous EVs were observed to lead to metastatic outgrowth and TME modifications (Hyenne et al., 2019).

How tumor and immune cells navigate through dense barriers like the ECM during invasion and infiltration has yet to be fully understood or agreed upon. In a captivating study using inducible zebrafish models with the mosaic expression of the oncogene HRAS^G12^ in different epidermal cell lineages, van den Berg et al. (2019), however, have captured the structural routes by which inflammatory cells traverse across the ECM to access pre-neoplastic cells. Through various imaging techniques, it was seen by the group that induction of HRAS^G12^ resulted in pronounced disruption of the skin architecture and led to the recruitment of macrophages and neutrophils. During this recruitment, correlative light and electron microscopy (CLEM) revealed that opportunistic macrophages and neutrophils were able to traverse into the epidermis through small preexisting openings in the ECM, which are used for immune surveillance under homeostatic conditions. It was further found that pre-neoplastic cells lying closer to these ECM weak spots possess a competitive advantage over those farther away, interacting with neutrophils more frequently and exhibiting faster growth rates (van den Berg et al., 2019).

Insight into the inflammatory and metastatic roles of the transmembrane Kunitz-type protease inhibitor 1 (SPINT1) in skin cutaneous melanoma (SKCM) has also been gained using adult and larval zebrafish models. Particularly, it was found that expression of SPINT1 was positively correlated with macrophage infiltration (though not neutrophil), and further that Spint1a deficiency is required at both cell-autonomous and cell-non-autonomous levels to alter immune surveillance and increase SKCM invasion (Gómez-Abenza et al., 2019). In zebrafish tumor xenografts, macrophages also directly interact with blood vessels and are suggested to enhance Vegfa-driven angiogenesis. Interestingly, it was found by this group that while ablation of zebrafish macrophages reduces xenograft vascularization, the ablation of neutrophils does not (Britto et al., 2018). Nevertheless, a *Kras*-driven melanoma zebrafish model has been used to show that neutrophils differentially express a number of pro-angiogenic genes and that neutrophil depletion significantly decreases blood vessel density and oncogenic liver sizes (Huo et al., 2019). Moreover, zebrafish neutrophils have been seen to be highly recruited to the microenvironment of *Kras*-transformed astrocytes with the aid of CXCR1, where they promote proliferation (Powell et al., 2018).

### Probing the Roles of Chemokine Signaling Axes in Cancer Progression

Chemokines are chemotactic cytokines that mediate immune cell migration in normal processes like development and tissue repair. Particularly, they affect target cells by binding to their respective G protein-coupled, chemokine receptors, which maintain an array of distinct and combinatorial functions through divergent pathways. Within the CXC subgroup of chemokines, CXCL12 ligand signaling through CXCR4 and CXCR7 receptors controls processes like cell leukocyte trafficking, migration, and hematopoiesis (Pawig et al., 2015). Opportunistic cancer cells, however, exploit this signaling axis by elevating their own CXCR4 levels to promote cell proliferation, angiogenesis, and ECM remodeling (Choi et al., 2014). Moreover, cancer cells have been found to home in on tissues with high levels of CXCL12 expression (Xue et al., 2017) and to increase their own secretion of CXCL12 to attract CXCR4-expressing stromal cells that can, in turn, assist with tumor development (Guo et al., 2016).

Tulotta et al. (2016) have shown that the CXCL12–CXCR4 signaling axis is functionally conserved across zebrafish and human cancer cells and that disruption of CXCR4 signaling *in vivo* impairs tumor invasion. Particularly, it was further observed by this group that interruption of Cxcr4b signaling in *cxcr4b* homozygous mutant zebrafish (*ody*) alters neutrophil adhesion and mobility (Tulotta et al., 2019). While neutrophils in wild-type larvae were seen to reduce their speed and interact with approaching tumor cell aggregates, Cxcr4b-deficient neutrophils in *ody* larvae showed no reduction in their speed and failed to infiltrate metastases. Intriguingly, expression of matrix metalloproteinase 9 (MMP9) in *ody* larvae was also found to be downregulated, which links to the group's previous work showing neutrophils contributing to the metastatic niche by conditioning the collagen matrix through physiological migration (He et al., 2012; Tulotta et al., 2019).

In breast cancer cell lines, it has been reported that the expression of CXCL12 and CXCR4 is regulated by the transcription factor POUF1 (Pit-1) and that knockdown of CXCR4 in Pit-1-overexpressing tumor cells significantly decreases tumor growth *in vivo* (Martinez-Ordoñez et al., 2018). Notably, the CXCL12–CXCR4 axis was found to be implicated in Pit-1-induced angiogenesis via autocrine and paracrine pathways (Martinez-Ordoñez et al., 2018). Moreover, CXCL12 has been seen to play an important role in the recruitment and transformation of macrophages into TAMs, which go on to cooperate with and increase the growth of Pit-1-overexpressing tumor cells when co-injected into zebrafish larvae (Seoane et al., 2019). Specifically, these findings are in accordance with those from Chia's group (Chia et al., 2018), who observed CXCL12–CXCR4 (Sdf1b-Cxcr4b) signaling to mediate macrophage infiltration and differentiation into microglia in zebrafish larvae modeling early glioma through the neuronal expression of the human oncogene AKT. It is worth noting that other intriguing work exploring the pro-tumoral contributions of microglia/macrophages in human glioma has also been reported and visualized in zebrafish (Hamilton et al., 2016), with the methodology described by Astell and Sieger (2017).

Still, there exist major immune signaling incongruences between zebrafish and human tumor cells that are relevant to tumor progression and the recapitulation of clonal heterogeneity. To address this issue in the context of leukemia, Rajan et al. (2019) have generated a humanized zebrafish model expressing the human cytokines CXCL12, stem cell factor (SCF), and granulocyte macrophage colony-stimulating factor (GM-CSF). In addition to increased survival and clonal representation among engrafted patient-derived acute myeloid leukemia cells, human hematopoietic stem and progenitor cells (HSPCs) were reported to survive in these transgenic larvae for up to 72 h post-injection (60 h longer than previous reports) and exhibit self-renewal and multilineage differentiation (Rajan et al., 2019). Aside from that, an effective method to induce mammal-zebrafish hematopoietic tissue chimeras have also been developed through xenotransplantation of murine bone marrow cells into zebrafish blastula (Parada-Kusz et al., 2018). Interestingly, active cell homing to hematopoietic tissues and response to bacterial infections were observed within these chimeras and suggestive of murine behavior.

## Evaluating Metastasis in Zebrafish

**Figure 2:**
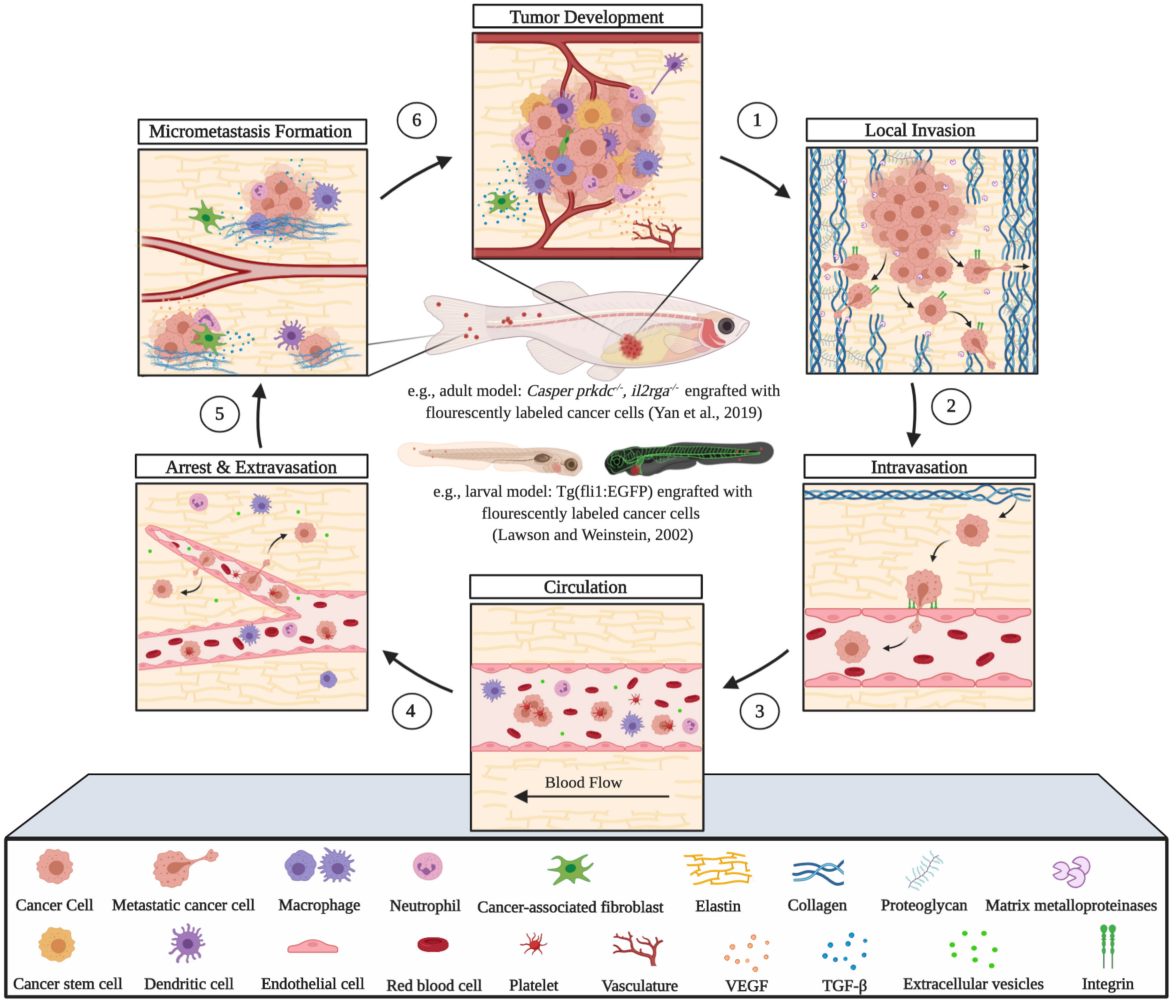
Zebrafish tumor models represent an alternative for studying the metastatic cascade by faithfully mimicking the tumor microenvironment (TME). Larval and optically clear adult zebrafish models permit every step of a malignant cell's journey to be visualized within the context of its TME.

The utility of zebrafish to probe metastatic events is poised largely on its optical clarity, which permits both close monitoring of individual cells and organismal structures in real time (Teng et al., 2013; Heilmann et al., 2015; Hill et al., 2018; Asokan et al., 2020). Combined with tools for genetic manipulation and fluorescent reporting (Lawson and Weinstein, 2002), every step in a malignant cell's journey, from invasion to colonization, can be interrogated *in vivo* (Figure 2) (Astell and Sieger, 2019; Osmani and Goetz, 2019). Our group has not only successfully established zebrafish-metastasis models but also demonstrated that inherent metastatic phenotypes of human cancer cells, as well as the genetic regulation of tumor metastasis, can be largely maintained in zebrafish (Teng et al., 2013).

### Metastatic Plasticity and Cooperative Invasion

Phenotype switching occurs frequently in response to different microenvironmental cues and has been linked to a number of clinically relevant processes like metastasis and drug resistance (Ahmed and Haass, 2018). Fluctuating expression of the transcription factor EWSR1-FLI1, for example, is reported to be a major source of phenotype plasticity in Ewing sarcoma cells, where EWSR1-FLI1^high^ states are characterized by active cell proliferation and EWSR1-FLI1^low^ states by invasion and metastasis (Franzetti et al., 2017). Particularly, decreased expression of EWSR1-FLI1 prompts changes in the actin cytoskeleton and causes a transition from cell–cell to cell–matrix adhesion, increasing metastasis and invasion *in vivo* (Franzetti et al., 2017). Melanoma phenotype switching between proliferative MITF^high^ states and invasive MITF^low^ states has also been explored in zebrafish and suggested to be induced by EDN3 among other microenvironmental factors (Kim et al., 2017). Interestingly, it has been found that tumor invasion can be led cooperatively between MITF^high^ and MITF^low^ cells, where inherently invasive melanoma cells switch from protease-independent to protease-dependent invasion while depositing ECM to aid the concurrent migration of non-invasive cells, similar to that of cancer-associated fibroblasts (CAFs) (Chapman et al., 2014). Human prostate cancer cell plasticity has additionally been tracked during early metastatic events in engrafted zebrafish. Here, metastases around caudal hematopoietic tissue were seen to respond to their microenvironment by upregulating epithelial–mesenchymal transition (EMT) and stemness markers, whose subsequent targeting suppresses metastatic growth (Chen et al., 2020). Finally, Yan et al. (2019) have impressively demonstrated their ability to track and identify human rhabdomyosarcoma migratory and proliferative cell states in zebrafish using photoconversion cell lineage tracing in an adult immunocompromised zebrafish strain. The group identified three phenotypically distinct rhabdomyosarcoma cell types *in vivo*: (1) bystander cells that showed no proliferation or movement over the course of 1 week, (2) actively proliferating cells, and (3) highly migratory cells. Interestingly, it was found that while proliferating cells could give rise to migratory cells, the opposite was not true (Yan et al., 2019).

### Dynamics of Dissemination

Both cell-intrinsic and microenvironmental factors influence the ways by which tumor cells disseminate to their secondary growth sites (Liu Q. et al., 2017). In early invasion, remodeling of the ECM, for example, can be achieved through the release of MMPs by stromal, inflammatory, or cancer cells (Lu et al., 2011). Although clinical studies using a broad range of MMP inhibitors have historically been unsuccessful, the exploration into the roles of MMPs and the pathways regulating their expression and activation (especially of MMP2 and MMP9) continues onward with the aid of zebrafish xenografts (Wyatt et al., 2017; Simbulan-Rosenthal et al., 2019; Wen et al., 2020). Integrin proteins have also attracted many researchers' attention. As principal cell-surface adhesion receptors that provide physical traction and carry out various signaling functions, integrins not only allow cancer cells to sense changes in their environment but also respond to it and alter it, for example, through the localization and activation of MMPs, promotion of angiogenesis (Desgrosellier and Cheresh, 2010), and activation of stem cells that in turn stimulate tumor progression (Yan et al., 2018). Accordingly, it has been reported that disrupting the expression or function of specific integrins like α3, αv, or α6 in cancer cells significantly reduces their ability to metastasize when transplanted into zebrafish (Li et al., 2015; Tanaka et al., 2019; Du et al., 2020). In one study combining intravital imaging with *Tg(fli1:EGFP)* transgenic zebrafish expressing GFP throughout their vasculature, the intravascular locomotion of breast cancer cells was seen to be reliant on β1-integrin-mediated adhesion to blood vessel walls (Stoletov et al., 2010). Moreover, it was demonstrated that β1-integrin was required for tumor cell VEGFA-mediated extravasation, which was associated with vascular remodeling (Stoletov et al., 2010).

Intriguingly, blood flow forces have also been investigated and found to contribute to the arrest, adhesion, and extravasation of circulating tumor cells in zebrafish larvae (Follain et al., 2018). Here, quantification of hemodynamic forces allowed for the identification of distinct regions of vasculature that vary in their conduciveness to cell arrest and endothelial remodeling (Follain et al., 2018). Additionally, it has been shown that vascular architecture itself is an exceptionally strong influencer of cell homing and arrest and that organ-specific extravasation is indeed affected by cell-specific differences and mediators like β1 integrin and myosin 1B (Paul et al., 2019a).

Aside from that, both CAFs and adipocytes have been reported to promote cancer invasion as well as proliferation in zebrafish tumor xenografts. In one study, coimplantation of color-coded cancer cells with either human-derived CAFs or normal healthy fibroblasts revealed the importance of CAFs in early-stage metastasis, particularly during initial intravasation and circulation, where it was observed that disseminating cancer cells stay in close association with CAFs (Liu C. et al., 2017). It was also demonstrated that stimulating normal healthy fibroblasts with growth factors like TGF-β and FGF-2 increased their metastatic capacity and rates of cancer metastasis (Liu C. et al., 2017). Specifically, these findings are in accordance with Sun and colleagues who also pointed to TGF-β signaling during their investigation into CAF-mediated cancer progression in zebrafish (Sun et al., 2019). Adipocytes also play noteworthy roles in metastasis, such as by supplying tumor cells with fuel and by structurally modifying the TME. To further elucidate these roles, Zhang et al. have employed various zebrafish models, including a *BRAF*^*V*600*E*^-driven transgenic model of melanoma alongside a MiniCoopR system that allows for a gene of interest to be coordinately expressed (Zhang et al., 2018). Here, the group found stromal adipocytes to drive melanoma invasion and growth through the direct transfer of lipids to melanoma cells via fatty acid transporter proteins and dysregulation of melanoma lipid genes (Zhang et al., 2018).

## Toward Adult Zebrafish Cancer Models and Zebrafish Patient-Derived Xenograft Models

Through the development of optically clear, immunodeficient zebrafish lines, features previously exclusive to larval models have gradually transcended into juvenile and adult stages. While optimization continues to be required, the advent of adult models nevertheless represents a milestone in zebrafish cancer research, opening the doors to larger and longer engraftment studies and more faithful recapitulation of the TME. Yan et al. (2019), for example, have reported generating *Casper* adult zebrafish (transparent) lacking T, B, and NK cells that effectively engraft a number of different human cancers when reared at 37°C. As the authors point out, combining this model with fluorescent reporter lines has the potential to expand the zebrafish toolkit and allow for even greater investigation into the TME's influence on cancer hallmarks and patient therapy responses.

Noticeably, an impetus to move zebrafish models toward personalized and precision medicine has been generated and is currently moving with considerable speed (Fior et al., 2017; Casey and Stewart, 2020; Costa et al., 2020a; Fazio et al., 2020). In regard to zPDX, both larval and adult models have demonstrated their remarkable potential to help guide clinical decisions; deciding which developmental stage to use, however, will likely depend on (1) the patient's timeframe, (2) the quantity of patient-derived material, (3) the number of therapeutic options planned to be tested, and (4) the tumor/TME aspects of interest. As mentioned by Xiao et al. (2020), larval xenograft assays may be the only option for patients with aggressive cancers that only have a few months to live or for those whose cancer treatment does not yet exist. In such cases, the employment of large scale drug screens and tumor radiosensitivity assays using zPDX may be pivotal to identify effective or novel therapies that would not be considered otherwise.

On the other hand, adult xenograft assays, which more accurately model the TME but require more patient-derived material and sacrifice a degree of throughput capacity, may be better suited for patients with slow-growing cancers whose treatment can be tailored, respectively over the years. Although much remains to be explored, a number of proof of concept and validation studies using zPDX have demonstrated the promising potential of zebrafish as a predictive pre- and co-clinical tool (Fior et al., 2017; Wang et al., 2019; Yan et al., 2019; Costa et al., 2020b; Usai et al., 2020). Most recently, we and other groups have also begun to tackle issues like engraftment rate and chemotherapy dose conversion, which must be refined to better establish the translational potential of zPDX.

## Conclusions and Future Perspectives

Although long underappreciated, the complexity and clinical significance of the TME have begun to be recognized through the facilitation of *in vivo* model systems. While zebrafish's investigative utility is largely poised on its inherent features, fashioning the system over the years through transgenic lines and engraftment techniques has significantly increased its probing potential. Nevertheless, the platform does maintain some limitations in its capacity to reliably model the TME. While certain shortcomings like organ complexity cannot be expected to be overcome, challenges surrounding pharmacokinetics, molecular signaling, and the effectiveness of gene knockouts and transgene insertions, can and ought to be further addressed in order to enhance zebrafish's predictive power and capacity to more faithfully recapitulate the TME. Specifically, Rajan et al. have shown the enormous potential of humanized models through the expression of human cytokines (Rajan et al., 2019); combining this model with adult zPDX (Yan et al., 2019), for example, would grant a more accurate representation of the microenvironmental factors and heterogeneity that are seen clinically. Of course, compounding model complexities is a balancing act that requires significant optimization. Encouragingly, advances in genome editing tools like CRISPR-Cas9, alongside recently developed methods like electroporation, which allow for spatiotemporal control over vector insertion (Callahan et al., 2018), have continued to expand our capacity to generate more complex and stable zebrafish lines. Moreover, efforts to standardize techniques and employ automated systems will help improve accuracy and reproducibility while safeguarding zebrafish's high-throughput capacity from time-consuming assays and validation studies. In closing, while zebrafish, like all model systems, is inherently limited to certain questions, exploration into its capacity as tool to investigate TME interactions and drive patient therapies forwards has only just begun.

## Author Contributions

YT conceived the original idea. RL wrote the manuscript with supervising from YT. CS and YT provided critical feedback and helped shape the manuscript. All authors contributed to the article and approved the submitted version.

## Conflict of Interest

The authors declare that the research was conducted in the absence of any commercial or financial relationships that could be construed as a potential conflict of interest.

## Abbreviations

CAFs: cancer-associated fibroblasts
ECM: extracellular matrix
EV: extracellular vesicles
HSPC: human hematopoietic stem and progenitor cell
MMP: matrix metalloproteinase
TAMs: tumor-associated macrophages
TANs: tumor-associated neutrophils
TNTs: tunneling nanotubules
TME: tumor microenvironment
zPDX: zebrafish patient-derived xenografts.
